# A Rare Complication of Composite Dual Mesh: Migration and Enterocutaneous Fistula Formation

**DOI:** 10.1155/2015/293659

**Published:** 2015-10-13

**Authors:** Ozgur Bostanci, Ufuk Oguz Idiz, Memet Yazar, Mehmet Mihmanli

**Affiliations:** ^1^Department of General Surgery, Sisli Etfal Training and Research Hospital, 34371 Istanbul, Turkey; ^2^Department of Plastic and Reconstructive Surgery, Sisli Etfal Training and Research Hospital, 34371 Istanbul, Turkey

## Abstract

*Introduction*. Mesh is commonly employed for abdominal hernia repair because it ensures a low recurrence rate. However, enterocutaneous fistula due to mesh migration can occur as a very rare, late complication, for which diagnosis is very difficult. *Presentation of Case*. Here we report the case of an enterocutaneous fistula due to late mesh migration in a mentally retarded, diabetic, 35-year-old male after umbilical hernia repair with composite dual mesh in 2010. *Discussion*. Mesh is a foreign substance, because of that some of the complications including hematoma, seroma, foreign body reaction, organ damage, infection, mesh rejection, and fistula formation may occur after implantation of the mesh. In the literature, most cases of mesh-associated enterocutaneous fistula due to migration involved polypropylene meshes. *Conclusion*. This case serves as a reminder of migration of composite dual meshes.

## 1. Introduction

Incisional hernia is among the most common complications of abdominal surgery. The incidence of incisional hernia is 10–15% and recurrence rate is 20–45% [1, 2]. Meshes are commonly used to minimize the recurrence of abdominal hernia repair. Polypropylene mesh is mostly preferred because its price is cheaper than composite mesh. All of the meshes have complications and they may occur even if years passed. Although late occurrence of mesh migration is the rarest of these complications, the diagnosis is very difficult. Most reports of enterocutaneous fistula due to mesh migration involve polypropylene mesh [3]. Here we describe a case of enterocutaneous fistula due to late migration of a composite dual mesh 4 years after incisional hernia repair.

## 2. Presentation of Case

**Figure 1:**
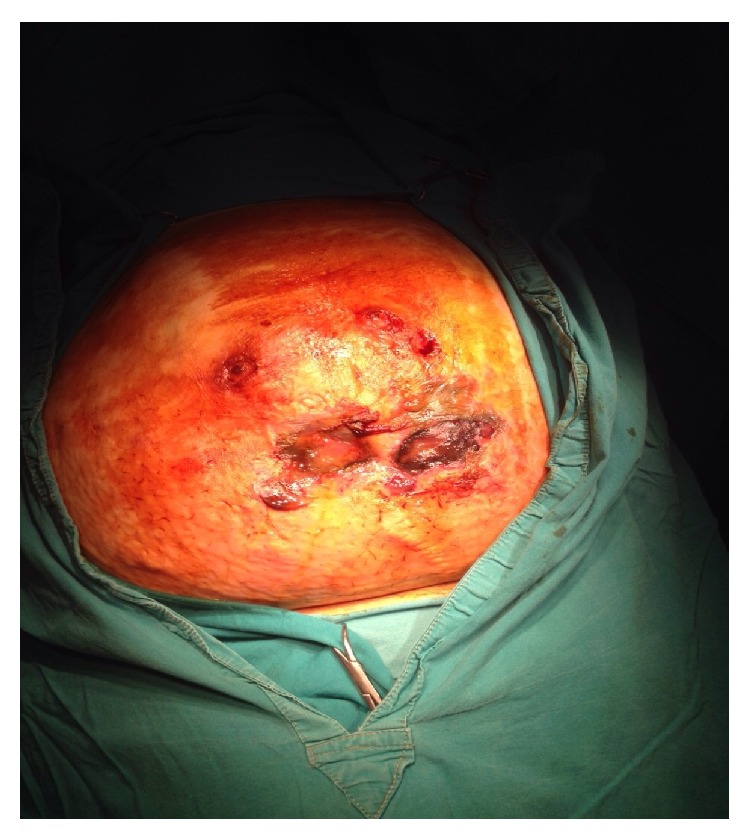
Preoperative photo of the patient.

Our patient was a mentally retarded, diabetic, 35-year-old male who underwent open hernia repair for a large umbilical hernia with the composite polypropylene and hyaluronate-carboxymethylcellulose mesh in 2010. Two years after surgery, he received treatment at an outpatient clinic for infection and necrosis of the umbilical skin, which included antibiotic administration and skin grafts. In November 2014, he presented at our emergency department because of abdominal pain and foul-smelling discharge from the abdominal wall. Upon admission, laboratory findings were as follows: white blood cell count of 13000 cells/microliter (reference range, 4400–9900) and serum C-reactive protein level of 135 mg/L (reference range, 0–5). Abdominal computed tomography (CT) with oral and intravenous contrast showed an ileal enterocutaneous fistula but revealed no mesh migration. Intraoperatively, we observed that the mesh migrated to the ileum from the subcutaneous tissue and there was an extensive subcutaneous inflammation with necrosis (Figure 1).

**Figure 2:**
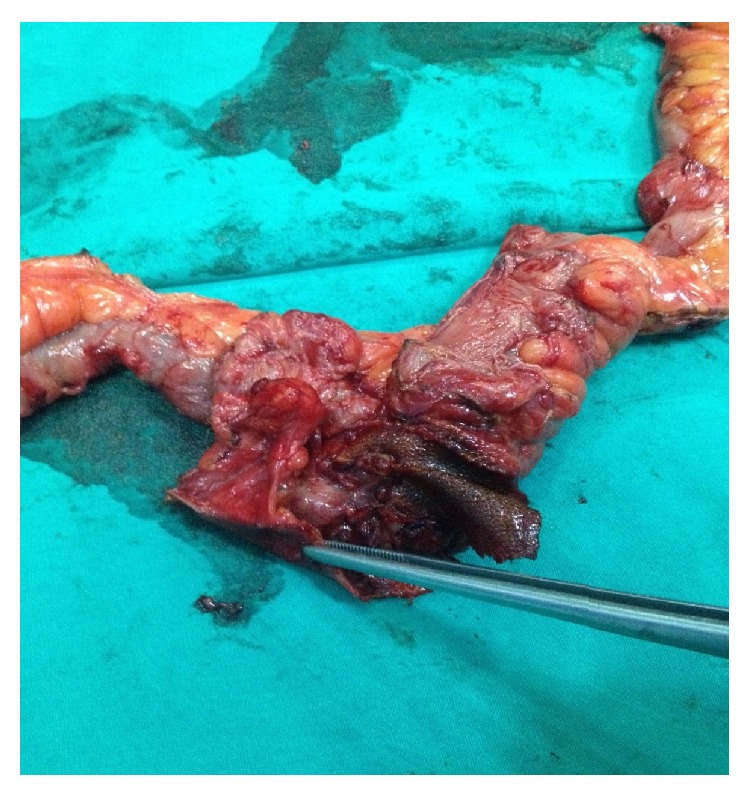
The pathological specimen and mesh migration to the intestine.

**Figure 3:**
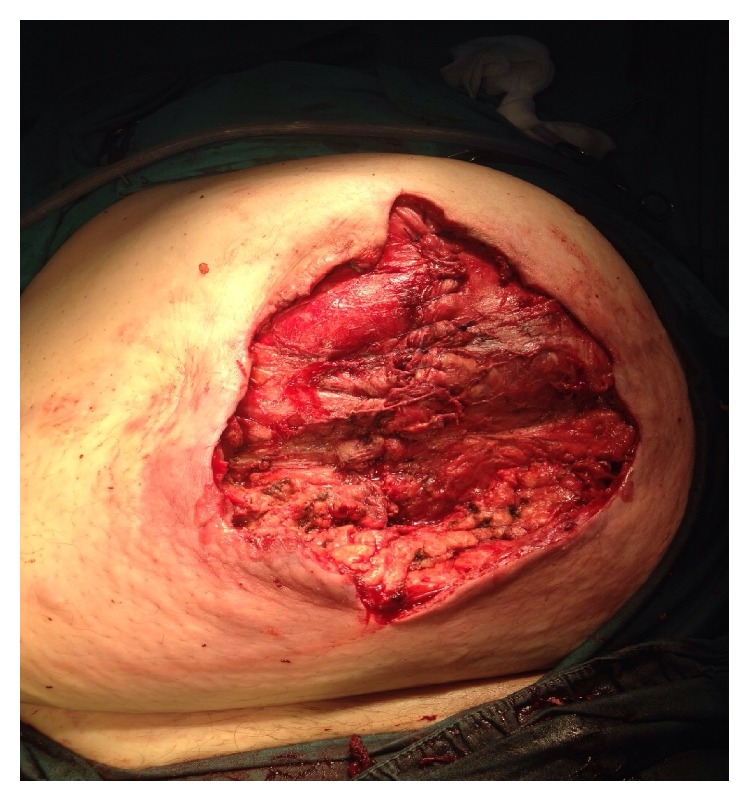
Postoperative photo of the wound.

We resected 100 cm of the ileum and debrided the necrotic skin with removal of subcutaneous tissue. The mesh was clearly observable on the pathological specimen (Figure 2). After debridement, the rectus fascia was mostly closed with the skin left open (Figure 3).

**Figure 4:**
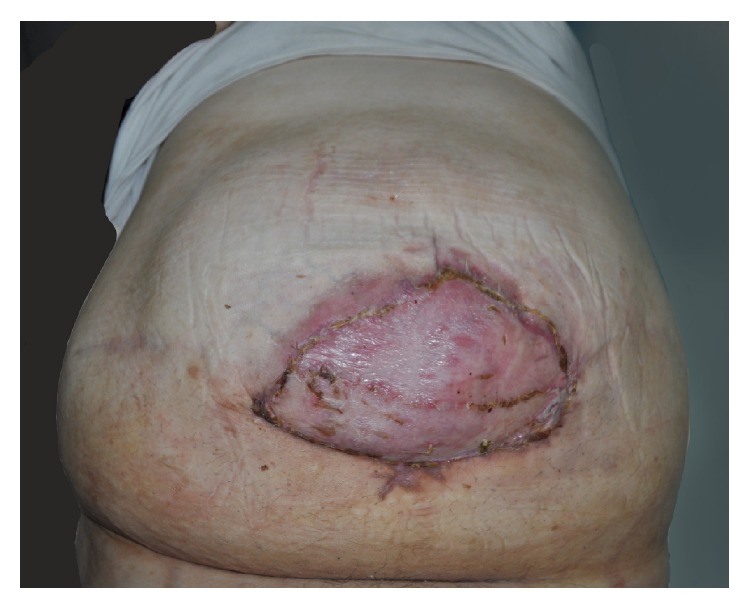
The wound after the skin graft.

The patient was observed daily and wet skin dressing was replaced appropriately. On postoperative day 17, inflammation was notably reduced and the patient was deemed sufficient for split-thickness skin grafting. Postoperatively, the patient was observed as an outpatient by our plastic surgery clinic (Figure 4).

## 3. Discussion

Obesity, advanced age, intra-abdominal ascites, pregnancy, malnutrition, chronic pulmonary disease, and corticosteroid are leading risk factors for the development of incisional hernia [4, 5]. The use of metallic meshes began in the 1940s but was subsequently abandoned in the 1970s due to wound complications [2]. According to several long-term retrospective studies, recurrence rates with simple suturing of incisional hernia repair reach 60%, whereas those of synthetic meshes are 30% [6–8]. However, mesh is a foreign substance, which may increase the risk of repair-related complications, including hematoma, seroma, foreign body reaction, organ damage, infection, mesh rejection, and fistula formation. Among these complications, mesh migration is relatively rare [9]. Mesh erosion and migration can present as acute intestinal obstruction, mass formation, bowel perforation, and chronic abdominal pain [10–13]. Ultrasound in the diagnosis of mesh migration is helpful, but limited in most cases. CT offers better images of mesh than ultrasound, although diagnosis of migration remains inadequate. Colonoscopy is the best diagnostic tool in the diagnosis of mesh migration to the colon [11–14]. Mesh migration may occur because of inadequate fixation of the mesh to the fascia or adequate fixation complicated by sliding via external forces and entry in the abdomen from points of least resistance. In addition, migration can occur acutely or in response to an inflammatory reaction to mesh erosion over a period of years [15–18].

Herrera was the first to report mesh migration in 1976, followed by a second case reported by Majeski of a woman who underwent incisional hernia repair with wire mesh and endured intestinal obstruction for 30 years afterward, before migration of the wire mesh to the intestine was confirmed [3, 19]. In our literature review there are a few composite mesh migration cases. Soler et al. compared composite mesh and Dacron mesh in an intraperitoneal rat study. At the end of the study the authors noticed that composite mesh migrated to the small bowel in one case [20]. In the other case, that is, a male patient who underwent ventral hernia repair, an enterocutaneous fistula was discovered intraoperatively and diagnosed by the surgeon as a sigmoidal mass. When the surgeon resected the mass, he noticed mesh migration to the sigmoid colon [21]. Also, Millas et al. reported a case of a composite mesh migration. A women who had umbilical hernia repair with composite mesh 2,5 years ago had lower abdominal pain. After barium enema, colonoscopy, and CT, she was operated on with the thought that the patient was having reactive changes from mesh placement. At the time of the operation surgeon identified that the mesh migrated to the sigmoid colon [22]. In our case, unlike most cases in the literature, the enterocutaneous fistula occurred after implantation of a composite dual mesh, as opposed to a polypropylene mesh.

## 4. Conclusions

Incisional hernia is among the most common complications of abdominal surgery and is commonly repaired using mesh. Many complications have been associated with mesh, although migration to the intestine and development of an enterocutaneous fistula are very rare. In the literature, most cases of mesh-associated enterocutaneous fistula due to migration involved polypropylene meshes. This case serves as a reminder of migration of composite dual meshes.

## References

[1] Mudge M., Hughes L. E. (1985). Incisional hernia: a 10 year prospective study of incidence and attitudes. *British Journal of Surgery*.

[2] Kingsnorth A., LeBlanc K. (2003). Hernias: inguinal and incisional. *The Lancet*.

[3] Herrera M. A., Hsia T. W., Becker D. R. (1976). Migration of teflon mesh from abdominal wall into large bowel. *New York State Journal of Medicine*.

[4] Townsend C. M., Beauchamp R. D., Evers M. B., Mattox K. L., Malangoni M. A., Rosen M. J. (2012). Hernias. *Sabiston Textbook of Surgery: The Biological Basis of Modern Surgical Practice*.

[5] Subramanian A., Clapp M. L., Hicks S. C., Awad S. S., Liang M. K. (2013). Laparoscopic ventral hernia repair: primary versus secondary hernias. *Journal of Surgical Research*.

[6] Burger J. W. A., Luijendijk R. W., Hop W. C. J. (2004). Long-term follow-up of a randomized controlled trial of suture versus mesh repair of incisional hernia. *Annals of Surgery*.

[7] Flum D. R., Horvath K., Koepsell T. (2003). Have outcomes of incisional hernia repair improved with time? A population-based analysis. *Annals of Surgery*.

[8] Luijendijk R. W., Hop W. C. J., van den Tol M. P. (2000). A comparison of suture repair with mesh repair for incisional hernia. *The New England Journal of Medicine*.

[9] Aziz F., Zaeemb M. (2014). Chronic abdominal pain secondary to mesh erosion into ceacum following incisional hernia repair: a case report and literature review. *Journal of Clinical Medicine Research*.

[10] Yilmaz I., Karakaş D. O., Sucullu I., Ozdemir Y., Yucel E. (2013). A rare cause of mechanical bowel obstruction: mesh migration. *Hernia*.

[11] Benedetti M., Albertario S., Niebel T. (2005). Intestinal perforation as a long-term complication of plug and mesh inguinal hernioplasty: case report. *Hernia*.

[12] Doctor H. G. (2006). Evaluation of various prosthetic materials and newer meshes for hernia repairs. *Journal of Minimal Access Surgery*.

[13] Gandhi D., Marcin S., Xin Z., Asha B., Kaswala D., Zamir B. (2011). Chronic abdominal pain secondary to mesh erosion into cecum following incisional hernia repair: a case report and literature review. *Annals of Gastroenterology*.

[14] Lange B., Langer C., Markus P. M., Becker H. (2003). Mesh penetration of the sigmoid colon following a transabdominal preperitoneal hernia repair. *Surgical Endoscopy*.

[15] Agrawal A., Avill R. (2006). Mesh migration following repair of inguinal hernia: a case report and review of literature. *Hernia*.

[16] Yolen S. R., Grossman E. T. (1989). Colonoscopic removal of a postoperative foreign body. *Journal of Clinical Gastroenterology*.

[17] Ojo P., Abenthroth A., Fiedler P., Yavorek G. (2006). Migrating mesh mimicking colonic malignancy. *The American Surgeon*.

[18] Jha A. K., Nijhawan S., Pokharna R., Nepalia S., Suchismita A. (2012). Colo-cutaneous fistula formation due to delayed mesh migration following lumbar hernia repair: colonoscopic diagnosis. *Tropical Gastroenterology*.

[19] Majeski J. (1998). Migration of wire mesh into the intestinal lumen causing an intestinal obstruction 30 years after repair of a ventral hernia. *Southern Medical Journal*.

[20] Soler M., Verhaeghe P., Essomba A., Sevestre H., Stoppa R. (1993). Treatment of postoperative incisional hernias by a composite prosthesis (polyester-polyglactin 910). Clinical and experimental study. *Annales de Chirurgie*.

[21] Nelson E. C., Vidovszky T. J. (2011). Composite mesh migration into the sigmoid colon following ventral hernia repair. *Hernia*.

[22] Millas S. G., Mesar T., Patel R. J. (2015). Chronic abdominal pain after ventral hernia due to mesh migration and erosion into the sigmoid colon from a distant site: a case report and review of literature. *Hernia*.

